# Association between controlling nutritional status (CONUT) and all-cause mortality in elderly hospitalized patients with acute exacerbation of chronic obstructive pulmonary disease: a retrospective cohort study

**DOI:** 10.3389/fnut.2026.1765476

**Published:** 2026-03-30

**Authors:** Shujiao Li, Mingzhe Wang, Jun Yan, Chengjun Ban

**Affiliations:** 1 Department of Respiratory Medicine, Dongzhimen Hospital, Beijing University of Chinese Medicine, Beijing, China; 2 The Third Affiliated Hospital of Beijing University of Traditional Chinese Medicine, Beijing, China

**Keywords:** chronic obstructive pulmonary disease, controlling nutritional status score, elderly people, malnutrition, mortality

## Abstract

**Objective:**

Nutritional status is a crucial modifiable factor that affects the prognosis of patients with chronic obstructive pulmonary disease (COPD). The CONUT score is a useful tool for comprehensively assessing nutritional status. This study aimed to investigate the relationship between the CONUT score at admission and the 3-year all-cause mortality rate among elderly patients hospitalized due to acute exacerbation of chronic obstructive pulmonary disease (AECOPD).

**Methods:**

This retrospective cohort study consecutively enrolled elderly patients hospitalized for AECOPD in the respiratory department of a tertiary hospital between 2013 and 2019. The CONUT score (based on serum albumin, total lymphocyte count, and total cholesterol) was calculated from initial admission laboratory results, categorizing patients into high-score (CONUT ≥ 5) and low-score (CONUT < 5) groups. The primary outcome was all-cause mortality over 3 years. Hazard ratios (HR) and their 95% confidence intervals (CI) were calculated using Cox proportional hazards regression models. Survival analysis was conducted using Kaplan-Meier curves, and dose-response relationships were explored using restricted cubic splines (RCS). Subgroup analyses were performed to assess the consistency of the association between a high CONUT score (≥5) and all-cause mortality.

**Results:**

This study included 931 patients with a median follow-up of 30 months. Patients with a high CONUT score (≥5) had a significantly higher risk of 3-year all-cause mortality compared to those with lower scores (adjusted HR = 2.62, 95% CI: 1.69–4.08, *P* < 0.001). RCS analysis revealed a non-linear association between CONUT score and mortality (*P* for non-linearity = 0.003). Subgroup analyses confirmed consistent associations across age, sex, smoking status, admission type, and prior AECOPD history.

**Conclusions:**

A higher CONUT score at admission is an independent risk factor for increased 3-year all-cause mortality in elderly hospitalized patients with AECOPD. This finding suggests that the CONUT score may serve as a simple and effective prognostic assessment tool for such high-risk patients, assisting in identifying individuals requiring enhanced nutritional support and management.

## Introduction

Chronic obstructive pulmonary disease (COPD) is a common chronic respiratory disorder characterized primarily by irreversible airflow limitation (1). Its prevalence and mortality rates increase significantly with age, causing the elderly to be particularly vulnerable to adverse outcomes (2, 3). Although traditionally viewed as a disease of the lungs, COPD is increasingly recognized as a systemic disorder associated with chronic inflammation, immune dysfunction, metabolic disturbances, and accelerated aging (4). These systemic effects contribute to frequent acute exacerbations and a generally unfavorable prognosis (5). Therefore, identifying effective predictive biomarkers for early detection would be beneficial for optimizing disease management and improving the prognosis of patients with COPD.

Nutritional status is a key determinant of health and survival in older adults (6, 7). Malnutrition is associated with exacerbated systemic inflammation, compromised immune function, and sarcopenia, factors which increase the frequency of disease exacerbations and the risk of mortality (7). The Controlling Nutritional Status (CONUT) score, calculated from serum albumin, total lymphocyte count, and total cholesterol, is a simple, composite tool for assessing nutritional and inflammatory status (8). It has demonstrated prognostic value in various chronic diseases. However, research on the predictive value of CONUT for long-term mortality in elderly hospitalized patients with COPD remains insufficient, and its clinical significance warrants further investigation.

Pathophysiologically, COPD progression involves not only airway inflammation but also intertwined systemic metabolic dysregulation (9). Malnutrition, inflammatory responses and immune dysfunction interact to accelerate disease progression and increase mortality (10). Therefore, investigating the prognostic potential of the CONUT score in elderly hospitalized patients with AECOPD may help explain the impact of nutritional status on outcomes and provide a basis for developing personalized nutritional intervention strategies.

In this context, we conducted a retrospective cohort study using data from a tertiary teaching hospital in Beijing, China. We aimed to evaluate the association between the CONUT score at admission and long-term all-cause mortality in elderly patients hospitalized for AECOPD. Through investigating the prognostic value of nutritional status, this study seeks to advocate for routine nutritional screening and intervention in elderly AECOPD inpatients, thereby aiming to improve their long-term survival.

## Methods

### Patients

We conducted a single-center, retrospective, cohort study. The study retrospectively analyzed the clinical data of elderly patients with AECOPD who were hospitalized in the general ward of the Department of Respiratory Medicine, Dongzhimen Hospital, Beijing University of Chinese Medicine from January 2013 to December 2019. This study was conducted in accordance with the Declaration of Helsinki and was approved by the Medical Ethics Committee of Dongzhimen Hospital, Beijing University of Chinese Medicine (Reference: 2022DZMEC-047-03).

The inclusion criteria were as follows: 1) Age ≥65 years; 2) The diagnosis of AECOPD meets the diagnostic criteria for AECOPD outlined in the GOLD; 3)For patients with multiple hospitalizations, we select the case records from their first admission. The exclusion criteria were defined as follows: 1) active tumor; 2) lack of clinical data; 3) patients lost to follow-up.

### Study variables

Demographic characteristics (age, sex, smoking history), prior history of AECOPD hospitalization (history of AECOPD hospitalization in the past 1 year), route of admission, comorbidities, laboratory test (blood routine test, biochemistry), and mechanical ventilation treatment were collected for all patients from the hospital database. The Charlson Comorbidity Index (CCI) was calculated for each patient based on their comorbidities. Additionally, length of hospital stay (LOS) of patients was recorded.

### Definitions of CONUT classes

The CONUT score was determined according to serum albumin (g/dL), total lymphocyte count (count/mm^3^), and total cholesterol (mg/dL) levels, as presented in Table 1. Patients were classified into four categories (normal, mild, moderate, and severe) based on the total CONUT score. For the primary analysis, patients were stratified into low (< 5) and high (≥5) CONUT groups according to a cutoff value of 5.

**Table 1:** Controlling nutritional status (CONUT) calculation.

Factors	Undernutrition status			
	Normal	Mild	Moderate	Severe
Albumin (g/dL)	≥3.5	3.0–3.49	2.5–2.9	< 2.5
Points	0.00	2.00	4.00	6.00
Total lymphocyte count (/mm^3^)	>1,600	1,200–1,599	800–1,199	< 800
Points	0.00	1.00	2.00	3.00
Total cholesterol (mg/dL)	>180	140–180	100–139	< 100
Points	0.00	1.00	2.00	3.00
Total CONUT score	0–1	2–4	5–8	5–12

CONUT, controlling nutritional status.

### Ascertainment of mortality

The primary outcome of this study was all-cause mortality, defined as death from any cause. Follow-up was conducted by combining hospital information system queries with telephone surveys. Follow-up commenced from the date of hospital admission. Patients were followed until death, loss to follow-up, or December 31, 2022, whichever occurred first. The primary outcome was defined as 3-year all-cause mortality. Based on their 3-year survival status, all patients were categorized into either the survival group or the death group.

### Sample size calculation

Sample size was calculated with reference to a published cohort study investigating nutritional risk and all-cause mortality in patients with COPD, which reported an adjusted hazard ratio (HR) of 2.47. This was a retrospective cohort study with the primary endpoint of 3-year all-cause mortality (10). The association between CONUT score and prognosis was analyzed using Cox proportional hazards regression. A two-sided type I error α = 0.05, power = 0.80, hypothesized HR = 2.47, and 10% loss to follow-up were applied. The minimum required sample size was 124.

### Statistical analysis

Descriptive statistics were used to summarize baseline characteristics, including medical history, comorbidities, and laboratory findings. Categorical variables are presented as numbers (percentages). The normality of continuous variables was assessed using the Kolmogorov–Smirnov test. Based on outcome status, patients were categorized into a survival group or a death group. Categorical variables were compared between groups using the chi–square test. Normally distributed continuous variables are presented as mean ± standard deviation (SD) and were compared using the independent samples *t*-test. Non-normally distributed continuous variables are expressed as medians (Q1, Q3) and were compared using the Mann–Whitney *U*-test. To address missing data, we first assessed the proportion of missing values for all continuous variables. Variables with a missing rate exceeding 25% were excluded from the analysis to ensure imputation stability. For variables with missing rates below 25%, missing values were imputed using the random forest algorithm (11, 12).

The CONUT score was calculated based on laboratory results obtained at admission, and patients were stratified into high (≥5) and low (< 5) CONUT groups. To assess the predictive value of a high CONUT score (≥5) for all-cause mortality, Cox proportional hazards regression models were used, with results presented as hazard ratios (HRs) and 95% confidence intervals (CIs). Potential confounders were selected based on previously published literature and clinical expertise, including demographic characteristics, lifestyle factors, and baseline health status (13).

Three models were constructed with sequential adjustments. Model 1 (unadjusted) included only the CONUT category to estimate the crude association with mortality. Model 2 (demographically adjusted) further controlled for age, sex, and smoking status to account for sociodemographic confounding. Model 3 (fully adjusted) additionally adjusted for medical history, comorbidities, and laboratory parameters to assess the independent predictive effect of CONUT after comprehensive confounding control. To examine the stability of the association and enhance interpretability, we employed a stepwise adjustment approach by progressing from a crude to a fully controlled estimate.

Survival across CONUT groups was compared using Kaplan-Meier estimates (log-rank test). The CONUT-mortality relationship was examined with restricted cubic splines and stratified analyses to test for effect modification. To explore the modifying effects of covariates on the predictive role of the CONUT score, stratified analyses were performed according to gender, age, smoking status, prior history of AECOPD, and ER admission. Results are reported as hazard ratios (HRs) and 95% confidence intervals (CIs), and interactions between groups were tested using interaction terms. All statistical analyses were performed using *R* software (version 4.4.1). A two-sided *P*-value of less than 0.05 was considered statistically significant. The STROBE checklist for this study is provided as Supplementary Table S1.

## Results

### Baseline characteristics

The study included 931 patients, comprising 519 males (55.75%) and 412 females (44.25%). The median age of the overall cohort was 78 years. According to CONUT, 449 (48.23%) patients had a normal nutritional status (CONUT 0–1), 402 (43.18%) had a mild (CONUT 2–4), 80 (8.59%) had a moderate (CONUT 5–8), and none had a severe impairment of nutritional status (CONUT 9–12). During a mean follow-up of 2.74y (minimum to maximum range, 0.02–3.0y), 154 of the patients (16.54%) died. The demographic and baseline clinical characteristics of the patients are detailed in Table 2.

**Table 2:** Baseline characteristics.

Characteristics	Total (*n* = 931)	Survival (*n* = 777)	Death (*n* = 154)	*P-*value
Age, M (Q_1_, Q_3_)	78.0 (72.0, 82.0)	77.0 (71.0, 82.0)	81.0 (78.3, 85.0)	< 0.001
Gender, *n* (%)				0.838
Female	412 (44.25)	345 (44.40)	67 (43.51)	
Male	519 (55.75)	432 (55.60)	87 (56.49)	
Smoking status, *n* (%)				0.185
Never	386 (41.46)	330 (42.47)	56 (36.36)	
Former	328 (35.23)	264 (33.98)	64 (41.56)	
Current	217 (23.31)	183 (23.55)	34 (22.08)	
Prior history of AECOPD, *n* (%)				0.04
No	696 (74.76)	591 (76.06)	105 (68.18)	
Yes	235 (25.24)	186 (23.94)	49 (31.82)	
Admission type, *n* (%)				< 0.001
Elective	723 (77.66)	626 (80.57)	97 (62.99)	
Emergency	208 (22.34)	151 (19.43)	57 (37.01)	
CCI score, M (Q_1_, Q_3_)	2.00 (1.00, 3.00)	2.00 (1.00, 3.00)	2.00 (1.00, 3.00)	< 0.001
CONUT score, M (Q_1_, Q_3_)	2.00 (1.00, 3.00)	1.00 (0.00, 3.00)	2.00 (1.00, 4.00)	< 0.001
CONUT Classe, *n* (%)				< 0.001
Normal-Mild	851 (91.41)	729 (93.82)	122 (79.22)	
Moderate-Severe	80 (8.59)	48 (6.18)	32 (20.78)	
Comorbidities				
Hypertension, *n* (%)				0.387
No	364 (39.10)	299 (38.48)	65 (42.21)	
Yes	567 (60.90)	478 (61.52)	89 (57.79)	
Diabetes, *n* (%)				0.955
No	715 (76.80)	597 (76.83)	118 (76.62)	
Yes	216 (23.20)	180 (23.17)	36 (23.38)	
Hyperlipidemia, *n* (%)				0.969
No	688 (73.90)	574 (73.87)	114 (74.03)	
Yes	243 (26.10)	203 (26.13)	40 (25.97)	
Coronary heart disease, *n* (%)				0.006
No	534 (57.36)	461 (59.33)	73 (47.40)	
Yes	397 (42.64)	316 (40.67)	81 (52.60)	
Atrial fibrillation, *n* (%)				< 0.001
No	835 (89.69)	709 (91.25)	126 (81.82)	
Yes	96 (10.31)	68 (8.75)	28 (18.18)	
Heart failure, *n* (%)				< 0.001
No	629 (67.56)	558 (71.81)	71 (46.10)	
Yes	302 (32.44)	219 (28.19)	83 (53.90)	
Stroke, *n* (%)				0.827
No	762 (81.85)	635 (81.72)	127 (82.47)	
Yes	169 (18.15)	142 (18.28)	27 (17.53)	
Chronic kidney disease, *n* (%)				< 0.001
No	857 (92.05)	727 (93.56)	130 (84.42)	
Yes	74 (7.95)	50 (6.44)	24 (15.58)	
Peripheral vascular disease, *n* (%)				0.751
No	815 (87.54)	679 (87.39)	136 (88.31)	
Yes	116 (12.46)	98 (12.61)	18 (11.69)	
Laboratory tests				
WBC (10^9^/L), M (Q_1_, Q_3_)	7.29 (5.80, 9.28)	7.20 (5.80, 9.13)	7.55 (5.93, 9.87)	0.22
RBC (10^12^/L), M (Q_1_, Q_3_)	4.29 (3.92, 4.63)	4.32 (3.96, 4.64)	4.11 (3.69, 4.59)	< 0.001
HGB (g/L), M (Q_1_, Q_3_)	132.00 (120.00, 143.00)	133.00 (122.00, 143.00)	125.00 (111.25, 139.00)	< 0.001
RDW (%), M (Q_1_, Q_3_)	13.30 (12.59, 14.00)	13.20 (12.50, 13.90)	13.80 (12.93, 14.90)	< 0.001
PLT (10^9^/L), M (Q_1_, Q_3_)	211.60 (174.00, 263.50)	212.00 (175.00, 263.00)	204.50 (166.25, 263.25)	0.424
Neutrophils (10^9^/L), M (Q_1_, Q_3_)	5.04 (3.71, 6.88)	4.95 (3.70, 6.73)	5.37 (3.83, 7.52)	0.023
Lymphocyte (10^9^/L), M (Q_1_, Q_3_)	1.30 (0.96, 1.70)	1.30 (0.98, 1.72)	1.22 (0.86, 1.56)	0.03
Eosinophilic (10^9^/L), M (Q_1_, Q_3_)	0.10 (0.05, 0.20)	0.10 (0.05, 0.20)	0.08 (0.03, 0.17)	0.015
Monocyte (10^9^/L), M (Q_1_, Q_3_)	0.57 (0.42, 0.73)	0.57 (0.42, 0.71)	0.57 (0.42, 0.81)	0.46
GLU (mmol/L), M (Q_1_, Q_3_)	6.25 (5.49, 7.79)	6.20 (5.48, 7.78)	6.43 (5.55, 8.21)	0.323
ALT (U/L), M (Q_1_, Q_3_)	24.00 (18.00, 31.00)	24.00 (18.00, 32.00)	22.00 (15.00, 28.93)	0.003
AST (U/L), M (Q_1_, Q_3_)	23.00 (19.00, 29.00)	23.00 (19.00, 29.00)	23.00 (18.00, 28.15)	0.644
LDH (U/L), M (Q_1_, Q_3_)	186.00 (158.95, 217.00)	185.00 (159.00, 214.00)	193.50 (155.40, 223.50)	0.506
Creatinine (μmol/L), M (Q_1_, Q_3_)	68.10 (57.15, 84.75)	68.10 (57.30, 83.80)	69.65 (56.62, 91.07)	0.477
Uric acid (μmol/L), M (Q_1_, Q_3_)	309.00 (242.00, 374.00)	307.00 (242.00, 372.00)	315.50 (243.00, 384.50)	0.526
TP (g/L), M (Q_1_, Q_3_)	68.80 (64.50, 73.20)	69.20 (65.00, 73.30)	66.75 (62.02, 71.55)	< 0.001
Albumin (g/L), M (Q_1_, Q_3_)	38.70 (35.20, 41.50)	39.00 (35.70, 41.70)	36.90 (32.52, 39.90)	< 0.001
Total cholesterol (mmol/L), M (Q_1_, Q_3_)	4.13 (3.50, 4.80)	4.18 (3.52, 4.82)	3.90 (3.34, 4.46)	0.002
HDLC (mmol/L), M (Q_1_, Q_3_)	1.19 (0.98, 1.45)	1.19 (0.98, 1.45)	1.16 (0.94, 1.45)	0.496
CRP (mg/L), *n* (%)				0.109
< 10	526 (56.50)	448 (57.66)	78 (50.65)	
≥10	405 (43.50)	329 (42.34)	76 (49.35)	
Mechanical ventilation, *n* (%)				< 0.001
No	872 (93.66)	741 (95.37)	131 (85.06)	
Yes	59 (6.34)	36 (4.63)	23 (14.94)	
LOS (days), M (Q1, Q3)	10.00 (8.00, 13.00)	10.00 (8.00, 13.00)	12.00 (9.00, 15.00)	< 0.001

CONUT, controlling nutritional status; CCI, Charlson comorbidity index; WBC, white blood cell count; RBC, red blood cell count; HGB, hemoglobin; RDW, red cell distribution width; PLT, platelet; Glu, glucose; ALT, alanine aminotransferase; AST, aspartate aminotransferase; LDH, lactate dehydrogenase; TP, total protein; HDLC, high-density lipoprotein cholesterol; CRP, C-reactive protein; LOS, length of hospital stay.

### Correlation between CONUT and mortality

**Figure 1:**
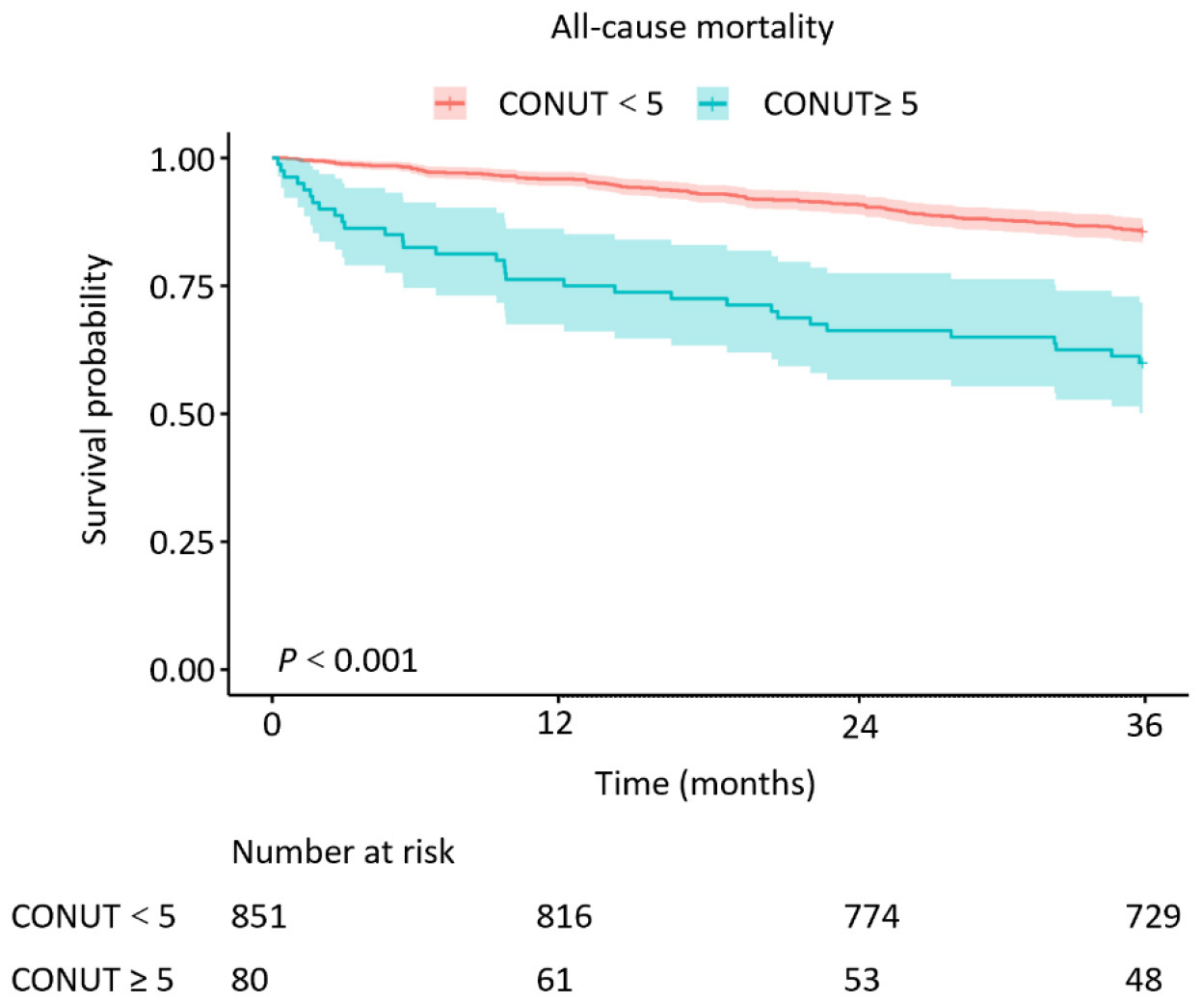
Kaplan–Meier survival rates of all-cause mortality in different CONUT groups of elderly inpatients with AECOPD.

**Figure 2:**
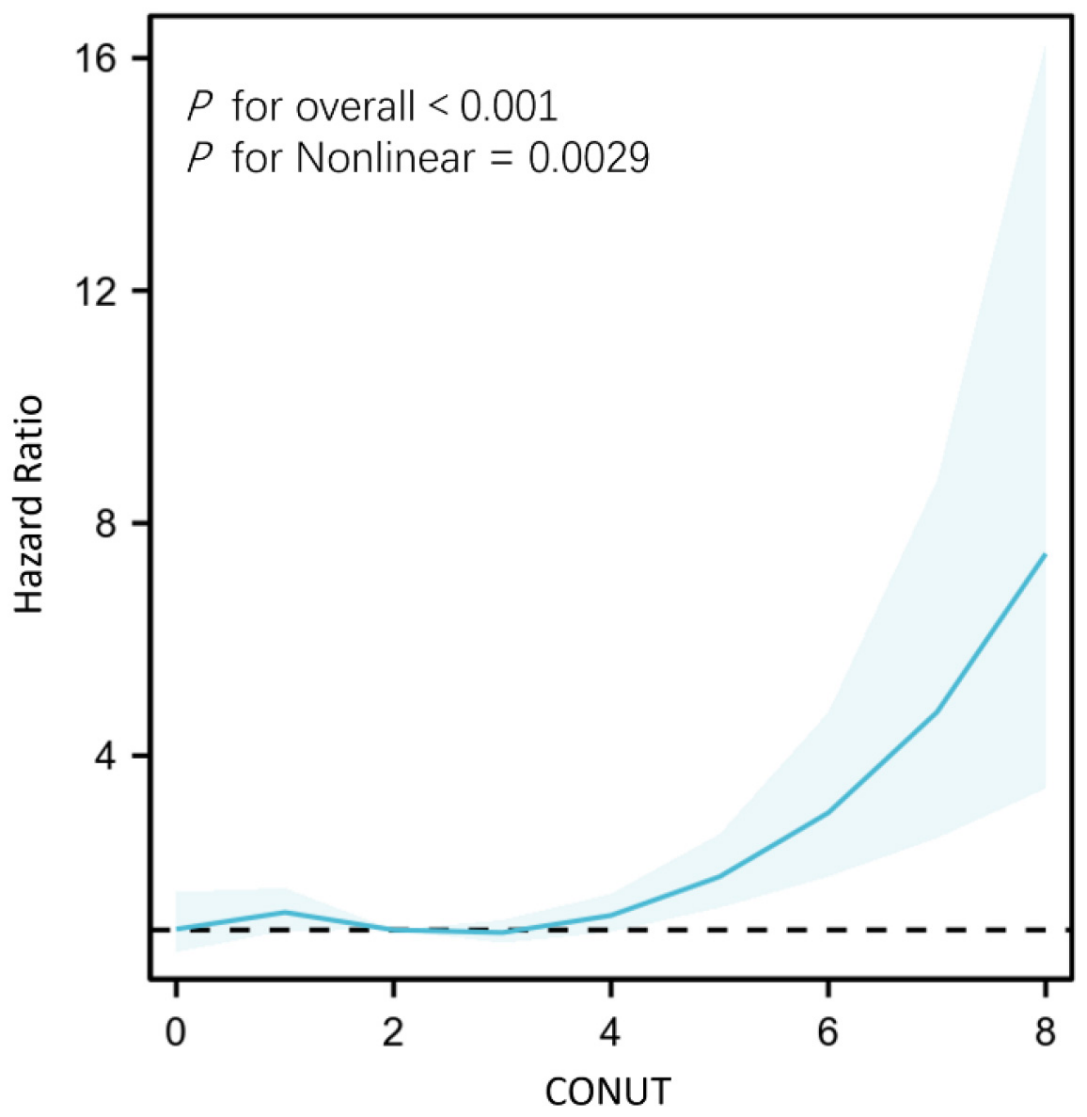
Restricted cubic spline regression of CONUT and all-cause mortality in elderly inpatients with AECOPD. Adjusted demographic characteristics, lifestyle factors, comorbidities and clinical characteristics. Adjusted demographic characteristics, lifestyle factors, and combined diseases and laboratory tests.

When treated as a continuous variable, higher CONUT score levels were associated with higher risk of all-cause mortality in all three models (Model 1: HR = 1.32, 95% CI: 1.22–1.44, Model 2: HR = 1.25, 95% CI: 1.14–1.36, Model 3: HR = 1.19, 95% CI: 1.07–1.32, all *P* < 0.001). As a categorical variable, a moderate-severe CONUT score was also associated with increased risk of all-cause mortality, with HR of 3.51, 3.01, and 2.62 in the Model 1 (95% CI: 2.38–5.19, *P* < 0.001), 2 (95% CI: 2.03–4.45, *P* < 0.001) and 3 (95% CI: 1.69–4.08, *P* < 0.001), respectively (Table 3). Sensitivity analysis using complete-case analysis produced essentially unchanged results. In the fully adjusted model (Model 3), the CONUT score as a continuous variable remained significantly associated with all-cause mortality (HR 1.34, 95% CI 1.17–1.54, *P* < 0.001), and the categorical CONUT showed a similar association (HR 3.51, 95% CI 1.94–6.37, *P* < 0.001; Supplementary Table S2). These findings support the robustness of the primary analysis. Furthermore, the survival analysis further validated our findings (Figure 1). Patients with high CONUT scores showed a lower survival rate. RCS analysis revealed a non-linear association between CONUT score and mortality (*P* for non-linearity = 0.003; Figure 2).

**Table 3:** Correlation between CONUT and all-cause mortality.

Variables	Model 1		Model 2		Model 3	
	HR (95%CI)	*P*-value	HR (95%CI)	*P*-value	HR (95%CI)	*P*-value
CONUT categorical						
< 5 score	ref		ref		ref	
≥5 score	3.51 (2.38–5.19)	< 0.001	3.01 (2.03–4.45)	< 0.001	2.62 (1.69–4.08)	< 0.001
CONUT score	1.32 (1.22–1.44)	< 0.001	1.25 (1.14–1.36)	< 0.001	1.19 (1.07–1.32)	< 0.001

Model 1: non-adjusted.

Model 2: adjusted for age(continuous), gender, and smoking status.

Model 3: adjusted for age(continuous), gender, smoking status, prior history of AECOPD, admission type, CCI score, hypertension, diabetes, hyperlipidemia, coronary heart disease, atrial fibrillation, heart failure, stroke, chronic kidney disease, peripheral vascular disease, WBC, RBC, HGB, RDW, PLT, neutrophils, eosinophilic, monocyte, GLU, ALT, AST, LDH, creatinine, uric acid, TP, HDLC, CRP, mechanical ventilation, and LOS.

CONUT, controlling nutritional status; HR, hazard ratio; 95%CI, 95% confidence intervals.

### Nonlinear and threshold effect analysis

RCS analysis demonstrated a significant non-linear relationship between the CONUT score and mortality risk (*P* for non-linearity = 0.003). Two-piece linear regression further revealed a significant threshold effect, with the inflection point at 3.6 points (likelihood-ratio test *P* = 0.003). Specifically, CONUT values below 3.6 were not significantly associated with all-cause mortality (HR = 0.94, 95% CI: 0.79–1.13, *P* = 0.525), whereas each one-point increase at or above 3.6 conferred a 57% increase in death risk (HR = 1.57, 95% CI: 1.21–2.05, *P* < 0.001) (Table 4).

**Table 4:** Threshold effect analysis of CONUT scores and all-cause mortality.

CONUT	Mortality	*P*-value
Model 1		
Straight-line effect	1.19 (1.08–1.30)	0.001
Model 2		
Knot (*K*)	3.6	
< K-segment effect 1	0.94 (0.79–1.13)	0.525
>K-segment effect 2	1.57 (1.21–2.05)	< 0.001
Log likelihood ratio tests		0.003

The inflection point (Knot) is located at CONUT = 3.6; the likelihood ratio test.

*P* = 0.003 indicates a significant threshold effect.

### Subgroup analyses

**Figure 3:**
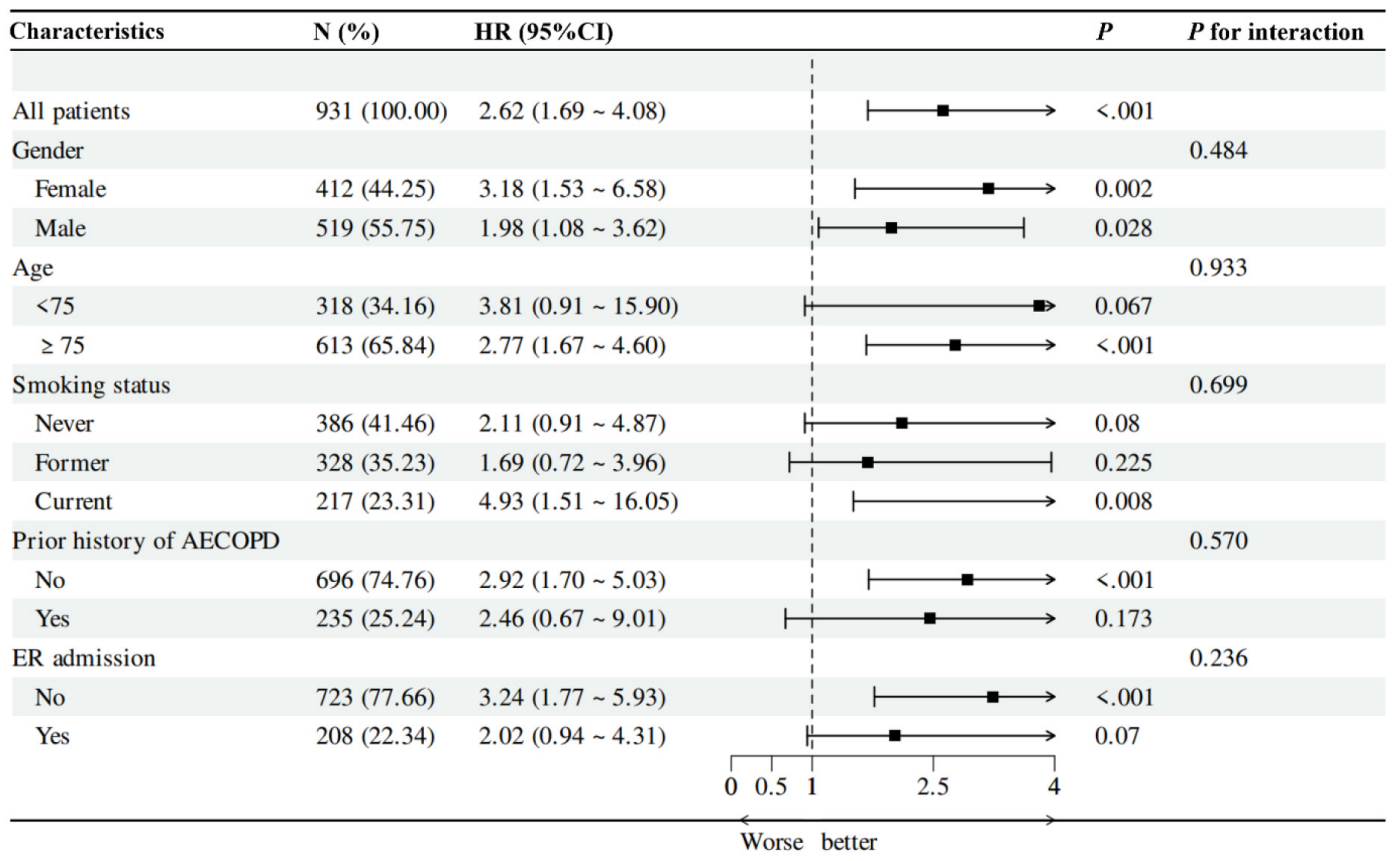
The correlation between all-cause mortality in subgroups of elderly inpatients with AECOPD.

Subgroup analyses were performed to assess the consistency of the association between a high CONUT score (≥5) and all-cause mortality (Figure 3). This association remained significant in females (HR = 3.18, 95% CI: 1.53–6.58, *P* = 0.002), males (HR = 1.98, 95% CI: 1.08–3.62, *P* = 0.028), patients aged ≥75 years (HR = 2.77, 95% CI: 1.67–4.60, *P* < 0.001), current smokers (HR = 4.93, 95% CI: 1.51–16.05, *P* = 0.008), those without prior AECOPD (HR = 2.92, 95% CI: 1.70–5.03, *P* < 0.001), and non-ER admissions (HR = 3.24, 95% CI: 1.77–5.93, *P* < 0.001). No significant interactions were detected between a high CONUT score and any subgroup variable (all *P* for interaction >0.05), suggesting the prognostic impact of a high CONUT score on all-cause mortality was consistent across these strata.

## Discussion

This study aimed to evaluate the association of the CONUT score with all-cause mortality among elderly inpatients with AECOPD. Our results identified the CONUT score as an independent predictor of mortality. This finding underscores the clinical relevance of assessing and addressing nutritional impairment in the management of these high-risk patients.

The relationship between nutritional status of COPD patients and their outcomes has gained increasing attention (14). Research indicates that malnutrition may aggravate systemic inflammation, impair muscle mass and function, and accelerate the decline of lung function (10, 15). These interconnected pathways collectively promote disease progression and elevate the risk of mortality.

The CONUT score is a composite, cost-effective tool for nutritional assessment, derived from serum albumin, total lymphocyte count, and total cholesterol. As a composite indicator, it is not only readily obtainable but also accurately reflects nutritional and immune status. In the present study, the CONUT score demonstrated that the prevalence of mild and moderate malnutrition among elderly patients hospitalized for AECOPD was 43.18% and 8.59%, respectively, which is consistent with previous reports.

Multiple studies have indicated the value of the CONUT score as a prognostic marker (16–20). In detail, Yuan et al. (46) conducted a systematic review and meta-analysis comprising 28 cohort studies (17 retrospective and 11 prospective), which established a significant association between higher CONUT scores and increased all-cause mortality in patients with heart failure (HR = 1.57, 95% CI: 1.35–1.83) (21). Furthermore, a prospective, multicenter cohort study conducted in Japan involving 2,773 non-dialysis CKD patients used Cox proportional hazards regression to confirm a significant link between the CONUT score and all-cause mortality (22). A cross-sectional study conducted in Italy by Lo Buglio et al. (14) enrolled 222 elderly patients with COPD (age ≥65 years). Using multivariate analysis, the study identified a CONUT score ≥5 as an independent risk factor for frequent acute exacerbations (≥2 episodes) in the past year (14). For long-term prognosis, a large-scale study based on the NHANES database found the CONUT score to be an independent predictor of mortality in patients with stable COPD (HR: 1.50, 95% CI: 1.18–1.91) (23).

However, prognostic data remain insufficient for elderly patients hospitalized for AECOPD, a particularly high-risk group, especially in Asian populations. To address this gap, we conducted a large-scale retrospective cohort study utilizing data from a tertiary teaching hospital in China. Aiming to leverage real-world data from elderly inpatients with AECOPD, this study involved follow-up for no less than 3 years to systematically analyze the impact of the CONUT score at admission on long-term all-cause mortality.

This study found that a higher CONUT score was significantly associated with increased all-cause mortality in elderly patients hospitalized for AECOPD. Specifically, patients with a CONUT score ≥5 had a significantly increased risk of all-cause mortality, an association that remained statistically significant after multivariable adjustment. This is consistent with prior evidence establishing CONUT as an independent risk factor for poor prognosis. Survival analysis confirmed that patients in the lower CONUT score group had a higher cumulative survival probability. Furthermore, analysis of the dose-response relationship using a RCS model revealed a monotonically increasing linear trend in mortality risk with higher CONUT scores, with no discernible threshold effect. Therefore, early identification and management of malnutrition could help improve prognosis.

As a comprehensive assessment tool, the CONUT score can be used to evaluate nutritional deficiency and immune dysfunction in patients with COPD, integrating key pathophysiological changes including malnutrition, metabolic disorders, and immune dysfunction. First, serum albumin levels reflect the nutritional status of the body and are involved in inflammation regulation (24, 25). Hypoalbuminemia not only indicates malnutrition but is also closely associated with systemic inflammation and oxidative stress (24); additionally, reduced albumin levels are significantly correlated with decreased muscle mass, impaired lung function, and an increased risk of acute exacerbation in patients with COPD (26–29). Second, total cholesterol reflects lipid metabolism and overall energy reserves. In elderly patients with COPD, chronic inflammation and hypercatabolism result in excessive cholesterol consumption, while malnutrition further limits its synthesis. Low total cholesterol levels are associated with a higher mortality risk and may impair the functions of innate and adaptive immune cells (e.g., by interfering with TLR signaling and immune synapse formation), thereby increasing susceptibility to infection and acute COPD exacerbations (30–33). Third, total lymphocyte

count is an important indicator of cellular immune function, which is gradually impaired with aging (immunosenescence) and persistent systemic inflammation in patients with COPD (34, 35). Lymphopenia leads to weakened immune defense and reduced disease resistance (36–38). Malnutrition further inhibits lymphocyte proliferation and function, forming a bidirectional vicious cycle: impaired immune function increases infection risk, while infection further exacerbates inflammatory responses and nutritional depletion (24, 39). Notably, these pathways do not operate in isolation. Chronic systemic inflammation, as a core driver, links malnutrition, metabolic dysregulation, and immune dysfunction through a feed-forward cascade: nutritional depletion impairs immune function, which may pre-dispose to persistent infection and amplified inflammation; in turn, unresolved inflammation accelerates catabolism and energy depletion, potentially exacerbating malnutrition (40–42). As a composite score, the CONUT index effectively captures this cumulative pathological burden, which suggests it can transcend the limitations of individual biomarkers. Collectively, these observations support the notion that the CONUT score reflects the integrated severity of nutritional, metabolic, and immune compromise, likely contributing to disease progression, recurrent acute exacerbations, and increased mortality in elderly patients with COPD.

An intriguing finding of this study is the identification of an inflection point at a CONUT score of 3.6, which is lower than the traditional cutoff of ≥5 defining moderate malnutrition. This discrepancy warrants careful interpretation. First, the traditional CONUT classification was primarily derived from community-dwelling or general hospitalized populations, which may differ substantially from elderly patients with AECOPD. The latter are characterized by heightened systemic inflammation, accelerated protein catabolism, and immunosenescence, all of which may render them more vulnerable to even modest nutritional deficits (34, 43). Second, the threshold of 3.6 was derived from a data-driven approach aimed at identifying the point at which the hazard for mortality begins to increase significantly. This statistical approach is designed to detect the earliest sign of risk escalation, whereas traditional cutoffs often represent more advanced stages of malnutrition. Clinically, this finding suggests that in elderly hospitalized AECOPD patients, nutritional risk may emerge at a lower CONUT score than previously recognized. Thus, a CONUT score exceeding 3.6 may serve as an early warning signal, prompting nutritional screening and intervention even before conventional criteria for moderate malnutrition are met. However, given that this threshold was derived from a single-center cohort, it requires external validation before clinical adoption. Future prospective studies should also examine whether interventions based on this lower cutoff translate into improved outcomes.

Our subgroup analyses further confirmed the robustness of the CONUT score as a prognostic indicator. This association remained consistent across subgroups stratified by age, sex, smoking status, and prior history of AECOPD hospitalization, with no significant interaction effects detected. Notably, the association appeared to be more pronounced in patients aged ≥75 years, current smokers, those without prior AECOPD, and non-ER admissions. In these subgroups, nutritional status might potentially act as a more sensitive marker of systemic vulnerability.

Several nutritional assessment tools have been employed to evaluate prognosis in COPD patients. The Geriatric Nutritional Risk Index (GNRI), derived from serum albumin and body mass index, was originally developed for hospitalized elderly patients. A recent NHANES-based study demonstrated that malnutrition defined by GNRI ( ≤ 98) was independently associated with increased all-cause mortality in COPD patients (10). The Mini Nutritional Assessment Short-Form (MNA-SF) is widely used in community settings, but its limited sensitivity (58.3%) against GLIM criteria in older COPD patients suggests that full diagnostic assessment may be warranted regardless of screening results (44). Compared with these tools, the CONUT score offers several practical advantages. First, it is entirely based on routine laboratory parameters, which are readily available at admission without additional measurements, eliminating subjective bias and enhancing reproducibility. Second, it provides a multidimensional assessment covering protein reserves, caloric status, and immune competence. Third, the CONUT score has been reported to have prognostic value for mortality prediction comparable to other nutritional indices in elderly COPD populations (45). No single nutritional tool is universally superior; selection should be guided by clinical setting and resources. While CONUT offers objectivity and convenience, tools like MNA-SF may provide additional insights in outpatient follow-up despite their sensitivity limitations.

### Strengths and limitations

This retrospective cohort study evaluated the association between the CONUT score at admission and all-cause mortality in elderly patients hospitalized for AECOPD. The use of real-world clinical data from a sizable sample enhances the reliability of the findings. However, several limitations should be acknowledged. First, as a single-center study, the generalizability of our findings may be limited to other populations or clinical settings. Therefore, future multicenter prospective studies are warranted to validate the prognostic utility of the CONUT score in diverse cohorts. Second, the CONUT score was assessed only at admission, representing a static measurement that does not capture dynamic nutritional changes during hospitalization. Future studies with serial assessments are warranted to provide a more comprehensive understanding of its prognostic implications. Thirdly, although multivariable adjustments were performed in this study, residual confounding factors may still exist, particularly those that were unmeasured. These include, for example, lung function, the use of long-term inhaled medications, nutritional support during hospitalization, and specific pharmacological treatments (e.g., corticosteroids). Future prospective studies should prioritize the consideration of these factors to further elucidate the independent prognostic value of the CONUT score. Given these limitations, the present findings should be interpreted cautiously and validated in future multicenter prospective studies.

## Conclusion

In conclusion, this study demonstrates that a high CONUT score is an independent predictor of 3-year all-cause mortality in elderly patients hospitalized with AECOPD. These findings underscore the importance of integrating routine nutritional assessment, using CONUT as a simple and effective screening tool, into the clinical management of this high-risk population to guide timely intervention. However, larger prospective studies with long-term follow-up are warranted to validate these conclusions.

## Data Availability

The original contributions presented in the study are included in the article/Supplementary material, further inquiries can be directed to the corresponding authors.
