# Supplementary material for: Association between controlling nutritional status (CONUT) and all-cause mortality in elderly hospitalized patients with acute exacerbation of chronic obstructive pulmonary disease: a retrospective cohort study
Source: Front Nutr. 2026 Mar 30;13:1765476. doi: 10.3389/fnut.2026.1765476 (PMC13070811; doi:10.3389/fnut.2026.1765476)
Supplement: Supplementary file 1 [file Table_1.docx]

**Table S1. STROBE statement**

|  | **Item No** | **Recommendation** | **Location in Manuscript** |
| --- | --- | --- | --- |
| **Title and abstract** | 1 | (a) Indicate the study's design with a commonly used term in the title or the abstract | Title section |
|  |  | (b) Provide in the abstract an informative and balanced summary of what was done and what was found | Abstract section |
| **Introduction** | | | |
| Background/rationale | 2 | Explain the scientific background and rationale for the investigation being reported | Introduction section |
| Objectives | 3 | State specific objectives, including any prespecified hypotheses | Introduction section |
| **Methods** | | | |
| Study design | 4 | Present key elements of study design early in the paper | Methods section: Patients |
| Setting | 5 | Describe the setting, locations, and relevant dates, including periods of recruitment, exposure, follow-up and data collection | Methods section: Ascertainment of mortality |
| Participants | 6 | (a) Give the eligibility criteria, and the sources and methods of selection of participants | Methods section: Patients |
| Variables | 7 | Clearly define all outcomes, exposures, predictors, potential confounders, and effect modifiers. Give diagnostic criteria, if applicable | Methods section: Study Variables and Ascertainment of mortality |
| Data sources/ measurement | 8 | For each variable of interest, give sources of data and details of methods of assessment (measurement). Describe comparability of assessment methods if there is more than one group | Methods section: Definitions of CONUT classes and Study Variables |
| Bias | 9 | Describe any efforts to address potential sources of bias | Methods section: Statistical analysis |
| Study size | 10 | Explain how the study size was arrived at | Methods section: Sample size calculation |
| Quantitative variables | 11 | Explain how quantitative variables were handled in the analyses. if applicable, describe which groupings were chosen and why | Methods section: Statistical analysis |
| Statistical methods | 12 | (a) Describe all statistical methods, including those used to control for confounding | Methods section: Statistical analysis |
|  |  | (b) Describe any methods used to examine subgroups and interactions | Methods section: Statistical analysis |
|  |  | (c) Explain how missing data were addressed | Methods section: Statistical analysis |
|  |  | (d) If applicable, describe analytical methods taking account of sampling strategy | N/A |
|  |  | (e) Describe any sensitivity analyses | Methods section: Statistical analysis |
| **Results** | | | |
| Participants | 13 | (a) Report numbers of individuals at each stage of study-eg numbers potentially eligible, examined for eligibility, confirmed eligible, included in the study, completing follow-up, and analyzed | Results section: Baseline characteristics |
|  |  | (b) Give reasons for non-participation at each stage | Methods section: Patients |
|  |  | (c) Consider use of a flow diagram | N/A |
| Descriptive data | 14 | (a) Give characteristics of study participants (eg demographic, clinical, social) and information on exposures and potential confounders | Results section: Baseline characteristics |
|  |  | (b) Indicate number of participants with missing data for each variable of interest | Methods section: Statistical analysis |
| Outcome data | 15 | Report numbers of outcome events or summary measures | Results section: Baseline characteristics |
| Main results | 16 | (a) Give unadjusted estimates and, if applicable, confounder-adjusted estimates and their precision (eg, 95% confidence interval). Make clear which confounders were adjusted for and why they were included | Results section: Baseline characteristics |
|  |  | (b) Report category boundaries when continuous variables were categorized | Methods section: Definitions of CONUT classes |
|  |  | (c) If relevant, consider translating estimates of relative risk into absolute risk for a meaningful time period | Results section: Baseline characteristics |
| Other analyses | 17 | Report other analyses done - eg analyses of subgroups and interactions, and sensitivity analyses | Results section: Subgroup analyses |
| **Discussion** | | | |
| Key results | 18 | Summarize key results with reference to study objectives | Discussion section |
| Limitations | 19 | Discuss limitations of the study, taking into account sources of potential bias or imprecision. Discuss both direction and magnitude of any potential bias | Discussion section: Strengths and Limitations |
| Interpretation | 20 | Give a cautious overall interpretation of results considering objectives, limitations, multiplicity of analyses, results from similar studies, and other relevant evidence | Discussion section |
| Generalizability | 21 | Discuss the generalizability (external validity) of the study results | Discussion section |
| **Other information** | | |  |
| Funding | 22 | Give the source of funding and the role of the funders for the present study and, if applicable, for the original study on which the present article is based | Funding section |

Give information separately for exposed and unexposed groups.

Abbreviation: N/A, not applicable.
