# Supplementary material for: Association between controlling nutritional status (CONUT) and all-cause mortality in elderly hospitalized patients with acute exacerbation of chronic obstructive pulmonary disease: a retrospective cohort study
Source: Front Nutr. 2026 Mar 30;13:1765476. doi: 10.3389/fnut.2026.1765476 (PMC13070811; doi:10.3389/fnut.2026.1765476)
Supplement: Supplementary file 2 [file Table_2.docx]

**Table S2.** Sensitivity analysis: Correlation between CONUT and all-cause mortality (complete case analysis).

|  |  | Model 1 |  |  | Model 2 |  |  | Model 3 |  |
| --- | --- | --- | --- | --- | --- | --- | --- | --- | --- |
|  |  | HR (95%CI) | *P* value |  | HR (95%CI) | *P* value |  | HR (95%CI) | *P* value |
| CONUT categorical |  |  |  |  |  |  |  |  |  |
| ＜5 score |  | ref |  |  | ref |  |  | ref |  |
| ≥5 score |  | 3.44 (2.24 - 5.30) | ＜0.001 |  | 2.84 (1.84 - 4.38) | ＜0.001 |  | 3.51 (1.94 ~ 6.37) | ＜0.001 |
| CONUT score |  | 1.28 (1.18 - 1.40) | ＜0.001 |  | 1.22 (1.11 - 1.33) | ＜0.001 |  | 1.34 (1.17 ~ 1.54) | ＜0.001 |

Notes: Model 1: Non-adjusted. Model 2: adjusted for age(continuous), gender, and smoking status. Model 3: adjusted for age(continuous), gender, smoking status, Prior history of AECOPD, Admission type, CCI score, Hypertension, Diabetes, Hyperlipidemia, Coronary heart disease, Atrial fibrillation, Heart failure, Stroke, Chronic kidney disease, Peripheral vascular disease, WBC, RBC, HGB, RDW, PLT, Neutrophils, eosinophilic, monocyte, GLU, ALT, AST, LDH, creatinine, uric acid, TP, HDLC, CRP, Mechanical ventilation, and LOS.

Abbreviations: CONUT, controlling nutritional status; HR: hazard ratio; 95%CI: 95% confidence intervals.
